# Does Vitamin C Deficiency Affect Cognitive Development and Function?

**DOI:** 10.3390/nu6093818

**Published:** 2014-09-19

**Authors:** Stine Normann Hansen, Pernille Tveden-Nyborg, Jens Lykkesfeldt

**Affiliations:** Department of Veterinary Disease Biology, Faculty of Health and Medical Sciences, University of Copenhagen, Ridebanevej 9, DK-1870 Frederiksberg C, Copenhagen, Denmark; E-Mails: stine.normann.hansen@gmail.com (S.N.H.); ptn@sund.ku.dk (P.T.-N.)

**Keywords:** vitamin C, cognition, oxidative stress, ROS, aging, development, stroke

## Abstract

Vitamin C is a pivotal antioxidant in the brain and has been reported to have numerous functions, including reactive oxygen species scavenging, neuromodulation, and involvement in angiogenesis. Absence of vitamin C in the brain has been shown to be detrimental to survival in newborn SVCT2(*−/−*) mice and perinatal deficiency have shown to reduce hippocampal volume and neuron number and cause decreased spatial cognition in guinea pigs, suggesting that maternal vitamin C deficiency could have severe consequences for the offspring. Furthermore, vitamin C deficiency has been proposed to play a role in age-related cognitive decline and in stroke risk and severity. The present review discusses the available literature on effects of vitamin C deficiency on the developing and aging brain with particular focus on* in vivo* experimentation and clinical studies.

## 1. Introduction

Increasing evidence is pointing to vitamin C (VitC) as an important redox homeostatic factor in the central nervous system, linking an inadequate dietary supply of VitC to negative effects on cognitive performance [1,2,3]. The brain displays comparatively high concentrations of VitC in particular during deficiency when most other organs are depleted [4,5,6], underlining an essential role of VitC in the brain. This has been further supported by findings of perinatal mortality and cerebral hemorrhages in newborn mice devoid the VitC transporter—thus, exposed to VitC depletion in the brain [7,8]—and by data showing reduced hippocampal volume and impaired spatial memory in guinea pig models of dietary induced VitC deficiency [1,9].

The only known clinical condition directly associated with lack of VitC is scurvy, representing the terminal and lethal collapse following prolonged and severe VitC deficiency (depletion) [10,11]. More recently, increased attention has been devoted the potential chronic effects of a suboptimal VitC status such as hypovitaminosis C—in humans defined as a plasma concentration below 23 µmol/L [12]—in disease development, e.g., impaired brain development [1,9], in multifactorial complexes of life-style associated diseases [13], and in neurodegenerative disorders [14,15,16,17].

Reports from cross-sectional population surveys have consistently estimated that at least 10%–15% of the adult population in the Western world suffers from suboptimal VitC levels/hypovitaminosis C [10,18]. This prevalence has been reported to be substantially increased in subgroups of particular risk, such as developing countries, communities of low socio-economic status, smokers, elderly, pregnant women, and children with poor nutritional status [19,20,21,22,23]. Thus, effects of VitC deficiency potentially affect millions on a global scale.

The present review outlines the functions of VitC in the brain and discusses putative effects of deficiency on cognitive ability based on findings from studies in animals and humans.

## 2. Vitamin C

VitC is a water soluble vitamin contributing as electron donor in several important biological reactions in the body. The active form of the vitamin, l-ascorbic acid, primarily exists as the monoanion ascorbate at physiological pH [24]. Most species are able to biosynthesize the micronutrient from glucose in the liver but higher-order primates including humans, as well as guinea pigs, and some bat, fish, and bird species are all dependent on an adequate dietary supply of VitC due to an evolutionary buildup of mutations and deletions in the gene encoding for l-gulono-1,4-lactone oxidase causing it to become non-functional [11]. Being unable to catalyze the final step in VitC biosynthesis, this renders the affected species entirely dependent on a dietary supply [25,26,27,28].

The uptake and distribution of VitC in the body is under close homeostatic control and primarily regulated by tissue specific sodium dependent vitamin C co-transporters (SVCT) 1 and 2, actively transporting VitC in exchange of sodium [29,30,31,32]. This results in a saturable plasma concentration (around 70 µM in humans [33]) that can only be increased via parenteral administration of VitC. Thus, the route of administration should clearly be considered when evaluating the potential effects of VitC intervention. In healthy individuals, the amount of the two-electron oxidation product of ascorbate, dehydroascorbic acid (DHA), represents only a small fraction of the total VitC pool due to efficient intracellular recycling to ascorbate and is generally considered to be of little importance in the overall homeostasis [34]. Within the body, VitC displays complex non-linear pharmacokinetics, as well as differential tissue distribution [31]. This includes the brain that is able to preferentially retain VitC at the expense of other tissues during chronic states of severe deficiency and to uphold concentrations 100-fold higher than, e.g., liver and kidney, which are readily depleted [5,6].

**Figure 1:**
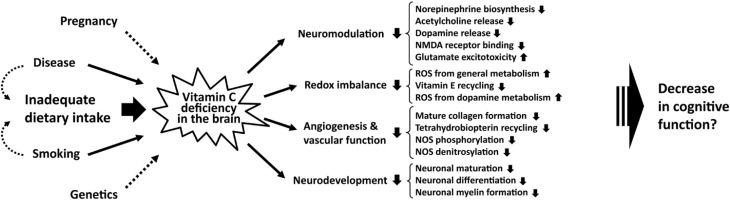
Proposed causes and consequences of VitC deficiency in the brain. Several risk factors for VitC deficiency have been identified, including disease, smoking, and inadequate dietary intake, but also pregnancy and genetics have been shown to affect VitC levels. Based on VitC’s involvement in important processes in the brain, there is reason to believe that these could be adversely affected by a deficiency. The functions of VitC are both related to its antioxidant function of upholding redox balance in the brain but also other important functions. These include modulation of the cholinergic, catecholinergic, and glutamergic systems of the brain, as well as the general development of neurons through maturation, differentiation and myelin formation. VitC is involved in several processes in the vascular system and hereby help maintain integrity and function of, e.g., nitric oxide synthase, which regulates vessel relaxation through production of nitric oxide. Abbreviations: NOS, nitric oxide synthase; ROS, reactive oxygen species; NMDA, *N*-methyl-d-aspartate.

The brain depends on the SVCT2 receptor to govern active VitC transport across the choroid plexus to the brain extracellular fluid and further on to neuronal cells [4]. Interestingly, neuroglia does not express the SVCT2 transporter, thus, are thought to rely on passive facilitated diffusion through GLUT-transporters for DHA transport; the DHA subsequently being reduced to ascorbate intracellularly [35,36]. In the brain, VitC is also regionally distributed with the hippocampus and the frontal and occipital cortex displaying high concentrations [36,37]. However, these apparent regional differences might—at least in part—be due to differences in neuronal density. Neurons have particularly high VitC concentrations (about 10 mM) compared to glia (about 1 mM) and, e.g., frontal-cortex and hippocampus both display high levels of neurons compared to other brain areas [38]. The SVCT2 expression also correlates with a 10-fold higher metabolism and, hence, reactive oxygen species (ROS) formation and, thus, antioxidant requirement in neurons [39,40,41,42]. Proposed causes and consequences of VitC deficiency in the brain are depicted in Figure 1.

## 3. The Functions of Vitamin C in the Brain

### 3.1. The Antioxidant Role of Vitamin C in the Brain

Due to a high level of poly-unsaturated fatty acids (PUFA), combined with high rates of cellular metabolism, the brain is particularly vulnerable to oxidative damage [43,44,45,46,47]. Several* in vitro* and* in vivo* experiments have supported a crucial role for VitC in the brain, both as a powerful antioxidant and scavenger of ROS, as well as a key factor in the recycling of other brain antioxidants, e.g., vitamin E (VitE) [5,6,48]. Mice born without functional SVCT2 transporters do not survive birth and display increased oxidative stress in the brain, as well as cerebral hemorrhages [7,8]. Redox-imbalances in the brain have been associated with ischemia-induced neurodegeneration [49,50,51,52] and in chronic diseases such as Alzheimer’s syndrome and Huntington’s chorea [14,15,16,17,53,54,55] as well as psychological disorders such as schizophrenia [56,57,58]. Collectively, these observations have led to the hypothesis that VitC plays a pivotal role in maintaining redox balance in the brain and subsequently that VitC deficiency leads to neuronal damage through processes involving increased ROS and oxidative stress [13]. Additionally, VitC has also been found to induce the expression of brain-derived-neurotrophic-factor—a component of several survival pathways—and may, thereby, contribute to the defense mechanisms of the brain [43].

### 3.2. Vitamin C as a Neuromodulator

VitC is known to participate in neuronal maturation and myelin formation, and also be involved in central nervous system signal transduction through neurotransmitters [59,60]. The dopamine (DA) receptor is involved in several different brain processes including pleasure, reward, motor control, and memory [61,62]. VitC supplies electrons for the dopamine-β-hydroxylase catalyzing the formation of norepinephrine from DA, and may provide neuroprotection from ROS and quinones generated by DA metabolism [63,64,65,66,67]. Thus, DA receptor activation has been shown to cause release of VitC in the brain and SVCT2 knock-out mice have a deficient adrenal catecholamine system and show increased adrenal cell apoptosis supporting an important role of VitC in DA homeostasis [68,69]. VitC has also been shown to induce the release of acetylcholine (ACh) and norepinephrine from synaptic vesicles of neurons, linking VitC to neuronal signal-transmission [70].

Another neuromodulatory role of VitC appears to be its involvement in presynaptic re-uptake of glutamate [2,71]. Here, VitC prevents excitotoxic damage caused by excess extracellular glutamate otherwise leading to hyperpolarization of the *N*-methyl-d-aspartate (NMDA) receptor and subsequent neuronal damage [40,71]. VitC has also been shown to inhibit the binding of glutamate to the NMDA receptor, in this way exhibiting a direct effect in the prevention of neuronal over-stimulation by glutamate [72,73].

Collectively, these findings imply that VitC is an important neuromodulator in the brain, and that depletion may have serious consequences for neuronal function and integrity.

### 3.3. Vitamin C in Angiogenesis

VitC’s function in collagen maturation is well recognized. Collagen is a primary component of supportive tissue and constitutes the basal membrane of blood vessels [7]. The final steps in the formation of mature triple helix collagen depend on VitC as electron donor in the hydroxylation of procollagen prolyl and lysyl residues [74,75,76]. VitC deficiency disrupts this collagen maturation leading to an impaired integrity of the vascular wall and ultimately resulting in hemorrhage—a cardinal symptom of scurvy—supported by cerebral bleedings in the SVCT2 knock-out mouse [7,8]. However, findings of unaffected levels of hydroxyproline in SVCT2 knock-out mice and in guinea pigs subjected to scorbutic VitC depletion suggests that VitC may even act as a *de novo* stimulator of collagen synthesis pointing towards more advanced functions besides that as an electron donor [8,77,78].

VitC’s angiogenic properties are further underlined by its apparent function as an electron donor for Fe2+-2-oxyglutarate-dependent dioxygenases catalyzing the hydroxylation of hypoxia inducible factor 1α (HIF-1α) [79,80]. Importantly, HIF-1α is involved in neuronal development, oxygen homeostasis and angiogenesis including vascular endothelial growth factor and erythropoetin [79,80,81,82]. Regulation of HIF is associated with VitC dependent hydroxylation and subsequent degradation, thus deficiency may increase HIF-1α levels hereby disturbing normal vascular development, likely to be particularly important in the growing fetus and in cases of regeneration following brain injury [51,52,83,84,85].

## 4. Vitamin C Deficiency and Cognitive Dysfunction

**Figure 2:**
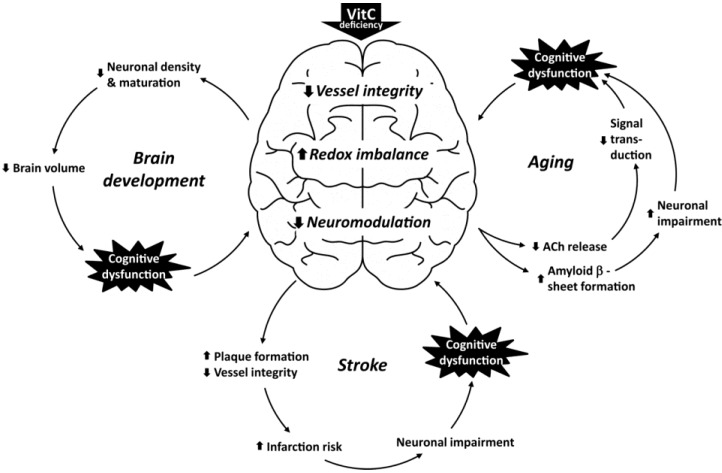
Vitamin C deficiency on cognitive function. The involvement of vitamin C in vessel integrity, redox balance and neuromodulation in the brain has prompted investigations into the effect of the vitamin on the developing brain, in aging and in stroke. In the developing brain, neuronal density and maturation is compromised by VitC deficiency, giving rise to decreased brain volume. In the aging brain deficiency affects ACh release and may impair cognitive function through reduced signal transduction but also through amyloid β deposition resulting in generation of reactive oxygen species and increased neuronal impairment in people suffering from Alzheimer’s disease. In stroke, VitC deficiency may result in decreased vessel integrity through, e.g., decreased NOS generation and impaired synthesis of mature collagen; potentially leading to increased plaque formation and incidence of stroke. Furthermore, an increase in infarct area may result from redox imbalance causing increased neuronal death.

The versatile roles of VitC related to the structural and functional integrity of the brain fuels the question of whether VitC deficiency may propagate cognitive dysfunction as suggested by findings in experimental animal models and reports from population surveys [1,3,15]. Of particular interests are the effects of VitC on the developing brain, age related neuronal degeneration, and induced brain injury (Figure 2).

### 4.1. Putative Consequences of Vitamin C Deficiency in the Developing Brain

The developing brain is particularly vulnerable to redox imbalances due to an undeveloped ROS defense system and a high metabolic rate, as well as having a high concentration of PUFA prone to oxidation [38,40,86]. While the establishment of the SVCT2 transporter as essential for VitC supply to the fetal brain and perinatal survival in mice demonstrates VitC as a key factor in brain development, the absolute lethality unfortunately also prohibits further assessment of functional consequences [7,8]. However, guinea pigs exposed to chronic but non-scorbutic VitC deficiency in early life showed significantly impaired spatial memory compared to sufficient counterparts and significant reductions in neuronal number in both the dentate gyrus and cornu ammonia of the hippocampus [1]. A later study of the effects of pre- and postnatal VitC deficiency in guinea pigs showed that 10%–15% reductions in hippocampal volume persisted into early adulthood (two months of age) and was unaffected by repletion [9]. None of the animals (sufficient or deficient) performed adequately in the Morris Water Maze to allow for conclusions to be drawn on cognitive performance, however, swimming parameters in the cued trial showed no apparent difference in locomotor ability between deficient and controls [9]. In gulo(*−*/*−*) mice unable to synthesize VitC, no effect on brain function could be detected following behavioral testing in the Y-maze, Morris Water Maze, and explorative activity monitor [87]. The apparent discrepancy between findings in mice and guinea pigs may be due to variations in trial conditions and/or specific responses to the imposed deficiency as well as species variations, e.g., prenatal* vs.* postnatal exposure and altricial* vs.* precocial offspring. However, a negative effect of chronic VitC deficiency on the hippocampus seems to be a consistent finding, at least in the guinea pig model, possibly leading to functional consequences, such as reductions in spatial memory [88,89].

Studies of young children exposed to intrauterine growth restriction (IUGR) or having a very low birth-weight have shown deleterious effects on cognition [90,91,92]. IUGR leads to a generalized malnutrition and, thus, does not asses the isolated effects of VitC deficiency. However, considering the high levels of VitC found in the young brain and the high metabolic rate during growth, a negative consequence of deficiency is not unlikely [93,94,95]. Tolsa* et al.* [92] reported that preterm babies (*n* = 14; gestational age: 32.5 ± 1.9 weeks) suffering from IUGR (birth weight: 1246 ± 299 g) displayed reduced brain volumes, particularly in the gray matter when compared to normal-weight preterm babies. At the calculated term date, the maintained significant volume reductions and, thus, no “catch-up” growth was observed. Furthermore, the reduction in brain volume was accompanied by a lower maturity by the Assessment of Preterm Infants’ Behavior-scoring. In a follow-up study of children born prematurely (*n* = 11, gestational age: median = 28 weeks) and with very low birth weights (median = 998 g; range: 840–1490 g), Isaacs* et al.* [91] reported long term effects on cognition at age 13.5 years (median age). When compared to controls, magnetic resonance-scans of prenatal children showed consistent atrophy of hippocampus and also atrophy of perioccipital white matter and corpus callosum as well as enlarged third and lateral ventricles. The neurological examination revealed that the study group had significantly impaired everyday memory by the Rivermead Behavioral Memory test, as well as lower numeracy skills in the Wechsler Objective Numerical Dimensions test [91].

The underlying pathogenesis of the anatomical and cognitive changes seen in IUGR is not completely understood, but it has been proposed that IUGR newborns have increased levels of oxidative stress. Biri* et al.* [96] measured oxidative stress markers in preterm IUGR babies (*n* = 13; gestational age: 33–34 weeks) and their mothers. Maternal blood, umbilical cord blood and placental samples were used to measure superoxide dismutase (SOD), glutathione peroxidase (GSH-Px), malondialdehyde (MDA), antioxidant potential (AOP), adenosine deaminase (ADA), catalase (CAT) and xanthine oxidase (XO). The umbilical cord blood from preterm babies showed significantly elevated oxidative stress in all markers, except GSH-Px and AOP, while the IUGR mothers differed significantly in all markers other than CAT, when compared to controls. The placental samples were also significantly changed in all aspects, except SOD and ADA [96]. In 29 preterm babies, Berger and coworkers [97] also reported high levels of F_2_-isoprostanes, but these were accompanied by high levels of VitC and did not correlate with potentially redox-active iron. Collectively, the above results suggest that the IUGR fetus is exposed to increased oxidative stress and tissue damage including the brain, which may partly explain the decreased cognitive functions and reduced brain volumes observed in these children later in life.

Another potential source of oxidative stress in the prenatal period is pre-eclampsia [98]. Several studies have concluded that maternal pre-eclampsia results in decreased cognitive performance in the affected children, when compared to children from healthy pregnancies [99,100,101,102]. Women with pre-eclampsia have been shown to have reduced levels of VitC and several studies have investigated a potential effect of VitC supplementation [103]. In a double-blind study, Chappell* et al.* [104] compared markers of oxidative stress in 79 high-risk women supplemented with VitC (1000 mg/day) and VitE (400 IU/day); 81 high-risk women offered placebo and 32 low-risk women not taking any supplements. VitC, plasminogen activator inhibitor (PAI)-2, and placenta growth factor concentrations were decreased in placebo group; and 8-epi-prostaglandin F2α, leptin, and the PAI-1/-2 ratio were increased in the placebo group compared with the low-risk group, whereas the vitamin supplement group displayed VitC, 8-epi-prostaglandin F2α, leptin, and PAI-1/-2 values similar to the low risk group. Thus, supplementation of high-risk women was associated with improvement in the biochemical indices of preeclampsia. However, studies have also reported no elaborate effects of supplementation with VitC and VitE in combination (1000 mg/day and 400 IU/day respectively) on the incidence of pre-eclampsia in high-risk women [105,106]. This might be due to the absence of VitC status as a defined inclusion criterion, hereby allowing for variation in the degree of plasma saturation and subsequent differential outcomes of supplementation [13]. Table 1 gives an overview of some of the studies done on oxidative stress and/or VitC in the developing brain, both* in vivo* and in clinical studies.

**Table 1:** Vitamin C, oxidative stress and brain development.

Species		Intervention	Measurements	Outcome	Reference
*In Vivo Studies*					
SVCT2(*−*/*−*) mice (ED: 18.5–19.5).		Dams: 0.33 g/L VitC in drinking water.	VitC content, MDA, F_2_-isoprostanes and F_4_-neuroprostanes in brain (cortex). Additional IHC.	The SVCT2(*−*/*−*) fetuses: Increased F_2_-isoprostanes (*p* < 0.001), F_4_-neuroprostanesincreased (*p* < 0.05), isoketal staining, apoptotic cells and hemorrhages. Decreased collagen-IV staining and VitC content (*p* < 0.001).	[7]
Dunkin Hartley guinea pigs (6/7 to 60/61days). Postnatal deficiency.		VitC in diet: 923 mg/kg or 100 mg/kg feed.	Asc, DHA, glutathione, MDA and SOD in brain. Quantitation of hippocampal neurons. Functional assessment in MWM.	Decreased performance in MWM (*p* < 0.05) and reduced number of neurons in hippocampus (*p* < 0.05) in VitC deficient animals.	[1]
Dunkin Hartley guinea pigs (GD: 18). Prenatal deficiency.		VitC prenatal: 900 mg/kg diet or 100 mg/kg diet. Postnatal: 750 mg/kg or 100 mg/kg.	Asc, DHA and MDA in brain. Hippocampal neurogenesis and volume. Functional assessment in MWM.	Significant and persistent lower hippocampal volume (*p* < 0.001).	[9]
Dunkin Hartley guinea pigs (2 days to 3 weeks).		VitC in diet: 1036 mg/kg or 36 mg/kg.	Asc, DHA, glutathione, SOD, MDA, α- and γ-tocopherol, protein carbonyls, 8-oxo-deoxyguanosine and base excision repair in brain.	VitC deficiency caused significant reductions in Asc (*p* < 0.001), DHA (*p* = 0.034), MDA (*p* < 0.001) and protein carbonyls (*p* = 0.003) and an increase in base excision repair (*p* = 0.014).	[5]
**Design and Subjects**		**Intervention**	**Measurements**	**Outcome**	**Reference**
*Clinical Studies*					
Cohort Studies	92 preterm children (7.86 ± 0.7 years, birth weight: 1475.13 ± 556.44 g) 40 age-matched controls.		Cognitive testing: Spatial pattern/Recognition, Intradimensional/Extra Dimensional Set-Shifting task, Tower of London task, Spatial Working Memory task, Spatial Memory Span task, and a Psychomotor screening.	Preterm children had decreased performance in Psychomotor test (*p* < 0.01), Recognition test (*p* < 0.01), Spatial Memory Span (*p* < 0.01), and Spatial Working Memory (*p* < 0.001)	[90]
	13 IUGR preterm infants (gestational age 33–34 weeks) and 12 controls.		Maternal blood, umbilical cord blood and placental samples: SOD, GSH-Px, MDA, AOP, ADA, CAT and XO.	All markers, except GSH-Px and AOP were elevated in umbilical cord blood. IUGR mothers differed significantly in all markers other than CAT. Placental samples were significantly changed in all markers, except SOD and ADA (*p* < 0.01 or less).	[96]
Controlled trials	Randomized clinical trial: 160 women in high risk for pre-eclampsia (16–22 weeks pregnant) and 32 controls.	VitC (1000 mg/day) and VitE (400 IU/day) or placebo.	Plasma VitC, PAI-2, placenta growth factor, 8-epi-prostaglandin F2α, leptin, PAI-1/2 ratio.	Vitamin supplemented: VitC, 8-epi-prostaglandin F2α, leptin, and PAI-1/-2 equal to controls; whereas placebo-treated displayed decreased VitC, PAI-2, and placenta growth factor and increased 8-epi-prostaglandin F2α, leptin, and PAI 1/-2 ratio.	[104]
	Randomized clinical trial: 283 women with high risk of pre-eclampsia (16–22 weeks pregnant).	VitC (1000 mg/day) and VitE (400 IU/day) or placebo.	PAI-1 and -2 measured every month until delivery. Pre-eclampsia assessed by the development of proteinuric hypertension.	VitC + E supplementation was associated with a decrease in the PAI-1/PAI-2 ratio (*p* = 0.015) and a significantly decreased risk of pre-eclampsia (*p* = 0.002).	[107]
	Double blind randomized clinical trial: 100 women in high risk of pre-eclampsia (14–20 weeks pregnant).	VitC (1000 mg/day) and VitE (400 IU/day) or placebo.	Incidence of pre-eclampsia.	No significant effect of VitC + E treatment.	[105]
	Double blind randomized clinical trial: 1365 women in high risk for pre-eclampsia (14–22 weeks pregnant).	VitC (1000 mg/day) and VitE (400 IU/day) or placebo.	Occurrence of pre-eclampsia defined as hypertension and onset of proteinuria.	Supplementation with VitC + E did not reduce risk of pre-eclampsia.	[106]

Abbreviations: VitC, vitamin C; VitE, vitamin E; Asc, ascorbate; DHA, dehydroascorbic acid; PAI, plasminogen activator inhibitor; MDA, malondialdehyde; GSH-Px, glutathione peroxidase; AOP, antioxidant potential; ADA, adenosine deaminase; CAT, catalase; XO, xanthine oxidase; SOD, superoxide dismutase; MVM, Morris Water Maze; IUGR, intrauterine growth restriction; IHC, immunohistochemistry; ED, embryonic day; GD, gestational day; SVCT, sodium-dependent vitamin C transporter.

### 4.2. Vitamin C and Aging

According to “The free radical theory of aging”, aging is the accumulated consequence of a lifetime of free radical assaults on the cells and macromolecules of the body [108]. In the aging brain, neurodegeneration has been associated with increased oxidative stress either through loss of electrons from the respiratory chain, inflammatory response or peroxide generation from β-amyloid [44,109,110].

VitC treatment (60–120 mg/kg intra-peritoneal; three to eight days) has been shown to attenuate reduced performance in elevated plus maze and passive avoidance test in seven-month-old Swiss mice, pointing towards VitC as an important factor in age-related cognitive decline [111]. In humans, meno-pause is associated with some degree of decline in cognition [112,113]. In ovariectomized rat-models of human meno-pause supplementation with VitC and VitE prevented deficits, hereby linking antioxidant status to the protection of cognitive function [114]. Notably, as both rat and mouse models are capable of synthesizing VitC, results should be interpreted with caution as the findings may not translate well to humans. In aging guinea pigs subjected to long term, non-scorbutic VitC deficiency (100 mg VitC/kg feed), no significant effects of age on biochemical markers in the brain were detected compared to controls (323 mg VitC/kg feed), and it was concluded that the age-related change in VitC status observed in several species is more likely related to maturation rather than aging *per se* [115,116].

In aging humans (*n* = 137; age: 66–90 years), plasma concentrations of VitC has been reported to be positively correlated with cognitive performance and plasma levels of VitC were significantly reduced in elderly suffering from different kinds of dementia [117,118]. This is supported by findings from a prospective cohort study from Department of Health/Medical Research Council Nutritional Programme in which participants (*n* = 921; ≥65 years) with the lowest VitC status displayed the poorest cognitive function, a finding which persisted when corrected for age, illness, social class, or other dietary variables [3]. In the Nurses’ Health study, long-term VitC and VitE supplementation prior to cognitive testing was significantly associated with better cognitive performance (*p* = 0.03) in women aged 70–79 years with a trend towards increasing performance with increased duration of use (*p* = 0.04) [118]. A similar effect on cognitive performance was found by Masaki* et al.* [119] in the Honolulu-Asia Aging study, where VitC or VitE supplementation was associated with a higher cognitive performance (OR: 1.25, 95% CI, 1.04–1.50). Furthermore, a protective effect of supplementation on vascular dementia (OR: 0.12, 95% CI, 0.02–0.88) and mixed/other dementia (OR: 0.31, 95% CI, 0.11–0.89) was found. However, other studies have not found an effect of self-reported VitC and/or VitE supplementation on age-related dementia [120].

The effect of VitC on cognition associated to aging has been extensively studied with regards to Alzheimer’s disease (AD) [121]—a disease already affecting many and predicted to increase in global prevalence [122]. Though the etiology of the disease is not completely elucidated, ROS and oxidative stress has been linked to disease progression [123]. As AD patients have been reported to have decreased levels of plasma VitC, investigations into the role of antioxidants including VitC in AD pathogenesis has been conducted [124,125]. In animal models of AD, VitC supplementation has been reported to reduce some of the cognitive dysfunction seen in control animals [126,127]. In a study of APP/PSEN1 mice, acute VitC administration (125 mg/kg intra-peritoneal, one hour prior to testing) significantly improved cognitive performance in both Y-maze and Morris Water Maze, albeit no significant effect was shown on either amyloid load, acetylcholine esterase (AChE) or oxidative stress markers. Likewise, an acute memory-enhancing effect of VitC has also been reported in other studies of age-dependent cognitive decline as well as in cognitive sound animal models [111,128].

In another set-up, 6 months old APP/PSEN1 mice exposed to four months of dietary VitC (1 g/kg diet) alone or in combination with high (750 IU/kg diet) or low (400 IU/kg diet) VitE showed decreased levels of F_4_-neuroprostanes and MDA—markers of lipid oxidation—in supplemented* vs.* controls while amyloid deposition was unaffected [126]. The low VitE + C treatment attenuated spatial memory deficits in APP/PSEN1 mice and improved performance in wild-type mice in the water maze. Interestingly, the high VitE + C treatment apparently impaired spatial memory compared to controls [126]. Likewise, VitC supplementation of drinking water (1333 mg/kg/day) to AβPP mice (six months old) for six months had no significant effect on the amyloid plaque load; however, an effect of VitC on cognitive function was noted [129].

Collectively, these data support the hypothesis that VitC can reduce cognitive decline in animal models while the exact mechanisms are yet to be disclosed. Studies of VitC supplementation during scopolamine-induced amnesia in mice have shown induction of AChE in the medial forebrain, pointing towards a role of VitC in the intricate regulation of cholinergic neurotransmission [130]. One potential mode of action has been suggested to be through the involvement of BH4:BH2 in the metabolism of mono-amine neurotransmitters—DA, norepinephrine and serotonin—as VitC maintains reduced biopterin status and may thereby indirectly regulate levels of neurotransmitters, known to be reduced in the memory deficits reported for AD patients [131].

In humans, VitC content of plasma and cerebrospinal fluid (CSF) in relation to cognitive decline in patients with mild to moderate AD (*n* = 32; mean age: 71 ± 7 years) was investigated [15]. A poor CSF/plasma VitC ratio was found to be a predictor of cognitive decline in AD in that each unit increase in CSF/plasma VitC ratio was associated with 1.1 units less point loss on Mini-Mental-Stale Examination and 2.7 units less loss on Alzheimer’s Disease Assessment Scale—Cognitive Section in 12 months [15]. Several studies have found that supplementation with VitC with or without VitE reduces the risk of AD [16,132], while others have failed to find this association [120,133]. In a study by Gray* et al.* [133] 2969 participants (≥65 years) were followed for a mean of 5.5 years and no significant effect of either VitC, VitE or multivitamin was associated with a decreased incidence of AD or dementia. In contrast, Engelhart* et al.* [16] found that supplementation lowered the risk of AD based on findings in 5395 participants (≥55 years) followed from 1990/93 to 1997/99. The reason for inconsistent findings in human studies may be found in the considerable variation in inclusion criteria including those of, e.g., plasma VitC status and definitions of “supplement user” between studies. Since ROS is thought to be a crucial part of the AD disease progression, it can be speculated that a consistently high VitC status acts in a preventive manner, while VitC supplementation *per se* is not a treatment for clinical AD. Thus, infrequent supplement users may not achieve the same benefits as individuals with consistent intake of adequate VitC. To more reliably investigate the possible preventive effect of VitC supplementation on AD development, well-designed randomized, controlled trials with adequate sample size and appropriate inclusion criteria are necessary [134,135]. In Table 2 is presented* in vivo* and clinical studies done on VitC and aging.

**Table 2:** Vitamin C and aging.

Species		Intervention	Measurement	Outcome	Reference
*In vivo studies*					
APP/PSEN1 and B6C3F1/J mice (6–10 months).		VitC in diet (1 g/kg) and high or low dose VitE (750/400 IU/kg).	Functional assessment, amyloid, F_4_-neuroprostanes and MDA.	Supplementation with VitC and low VitE decreased markers of oxidative stress in transgenic mice (*p* < 0.05). Improvement of MWM performance was seen in low VitE group (0.05 > *p* < 0.001).	[127]
Swiss mice (3 and 7 months).		IP injection of 60 and 120 mg/kg VitC for three or eight consecutive days.	Elevated plus maze, passive avoidance test.	Treatment improved performance in young animals (*p* < 0.05) and reversed performance deficits in old animals (*p* < 0.05).	[111]
Dunkin Hartley guinea pig (3–9 months and 36–42 months).		Diet containing 325 mg VitC/kg or 100 mg VitC/kg.	VitC, MDA, glutathione, 8-oxodG and SOD in brain. SVCT2 mRNA expression in brain.	Deficiency did not cause significant changes in oxidative stress markers but aging *per se* showed a significant effect (*p* < 0.05). No detectable effect on SVCT mRNA expression in deficient.	[116]
AβPP mice (6–12 months).		1333 mg/kg/day VitC in drinking water.	IHC for anti-Aβ, Western blot, Aβ-ELISA, OxyBlot, glutathione, functional assessment by MVM and elevated plus maze.	VitC prevented some behavioral abnormalities in AβPP mice (0.05 > *p* < 0.02), down-regulated amyloid (*p* < 0.05), significant difference of Aβ42/Aβ40 ratio (*p* < 0.02) and increased in synaptophysin (*p* < 0.05). Phosphorylated tau was decreased (*p* < 0.05).	[129]
Female ovariectomized Wistar rats (80 days).		VitE (40 mg/kg) and VitC (100 mg/kg) IP once daily for 30 days.	MWM, open field test.	Vitamin C + E treatment prevented deficits in reference memory in MWM (0.01 > *p* < 0.05).	[114]
Swiss mice (3 months).		VitC (60 mg/kg) IP injection of for three consecutive days.	Elevated plus maze and passive avoidance.	VitC injection reversed amnesia induced by scopolamine (0.4 mg/kg) and diazepam (1 mg/kg) (*p* < 0.05).	[111]
B6C3F1/J mice (12 weeks).		VitC (125 mg/kg) IP.	Behavioral testing, MDA and Asc content in cortex, AChE activity, brain glutathione.	VitC treatment reversed some of the memory deficits induced by scopolamine (1 mg/kg IP) (0.05 > *p* < 0.001) and increased medial forebrain AChE acticity (*p* < 0.001).	[130]
CD1 mice (16 months).		Oxiracetam (62.5/125/250 mg/kg), VitC (50/100/200 mg/kg), VitC (125 mg/kg) + oxiracetam (100 mg/kg) IP for three consecutive days.	Light-dark aversion test.	VitC alone or in combination with oxiracetam significantly reduced scopolamine-induced (0.25 mg/kg IP, day 4) amnesia (*p* < 0.01).	[136]
Dunkin Hartley guinea pigs (3–9 and 8–14 months).		Diet with 325 mg/kg VitC.	VitC in brain and CSF.	Concentrations of VitC significantly increased in CSF with age (*p* < 0.05). Elevated Asc oxidation ratio in young compared to old animals (*p* < 0.05).	[115]
Dunkin Hartley guinea pigs (3–9 and 36–42 months).		Diet with 325 mg VitC/kg or 100 mg VitC/kg.	VitC in CSF and 8-oxodG, MDA, glutathione and SOD in brain.	No effect was observed besides on VitC concentration in brain and CSF in deficient animals.	[116]
**Design and Subjects**		**Intervention**	**Measurements**	**Outcome**	**Reference**
*Clinical Studies*					
Cohort studies	12 AD or dementia patients (71 ± 11 years) and healthy controls (35 ± 5 years).		Blood samples of VitC and DHA.	Dementia and AD patients had significantly lower Asc and DHA levels (*p* < 0.001).	[137]
	Patients (*n* = 134) (AD, vascular dementia or Parkinson’s) and 58 matching controls.		Plasma content of: α-carotene, β-carotene, lycopene, VitA, VitC, VitE and TAC.	VitC was significantly lower in AD (*p* < 0.001), vascular dementia (*p* < 0.001) and Parkinson’s disease with dementia (*p* < 0.01).	[117]
	Prospective cohort study: 633 participants age ≥65 years.	Direct inspection of ingested supplements (two weeks of base-line). Participants were followed for a mean of 4.3 years.		None of the VitE or VitC users developed AD despite a predicted incidence of 3.9 and 3.2, respectively (*p* = 0.04).	[132]
	Nurses’ Health study: 14,968 women age 70–79 in 1995–2000.	Semi-quantitative questionnaire on lifestyle, supplemental use and medical history biennially from 1980.	TICS, 10-word list, immediate and delayed recall, verbal fluency, digit span backwards test.	Long-term VitC + E supplementation was associated with better cognitive function (*p* = 0.03) and a trend toward better performance (*p* = 0.04)	[118]
	The Honolulu-Asia Aging Study: 3385 men age 71–93 years.	Questionnaires on vitamin supplementation in 1982/1988.	Assessment of cognitive performance by CASI in 1991–1993.	VitC and/or VitE supplementation decreased the incidence of vascular (OR: 0.12) and mixed/other type dementia (OR: 0.31) and was associated with a higher cognitive performance (OR: 1.25).	[119]
	The Rotterdam Prospective Study: 5395 participants age ≥55 years in 1990–1993	Interview of dietary intake of VitC, VitE, β-carotene, supplements, educational level, *etc.*	Clinical examination and MMSE, GMS, CAMDEX in 1993–1994 and 1997–1999.	High dietary intake of VitC and VitE may lower the risk of Alzheimer’s disease. RR = +0.82/standard deviation increase in VitC intake.	[16]
	Prospective cohort study: 32 patients with mild to moderate AD age 71 ± 7 years.	Physical examination.	ADAS-cog, MMSE, CDR and geriatric depression. CSF and blood samples at baseline.	CSF/Plasma VitC content predicted cognitive decline partially due to a compromised blood brain barrier integrity.	[15]
	Nurses’ Health Study: 16,010 women age ≥70 years in 1995–2000.	Food-questionnaire in 1980 and expanded version in 1984, 1986, and every four years thereafter. FRAP assessment.	TICS scores and ten word list, global composite scores, East Boston Memory test on three occations.	No significant association between FRAP scores and cognitive function, when adjusted for confounders.	[138]
	Adult Changes in Thought Prospective Study: 2969 participants age ≥65 years.	Self-reported VitC, VitE or multivitamin supplement. Participants were followed for a mean of 5.5 years.	Health and lifestyle parameters (e.g., BMI, smoking and alcohol consumption) CASI score every second year.	Neither VitC, VitE nor multivitamin use was associated with a decreased incidence of AD or dementia.	[133]
	Prospective cohort study: 137 elderly age 66–90 years.	Nutritional data collected in 1980 and 1986.	Cognitive evaluation in Logical Memory, Abstraction and Visual Reproduction trials in 1986.	Plasma concentrations of VitC were positively correlated with Rey-Osterrieth Copy test performance and Visual Reproduction (*p* < 0.05).	[139]
	Prospective cohort study: 921 elderly age ≥65 years.	A one week food diary or interviews to quantify consumer habits. Participants were followed for 20 years.	Medical examination including Hodkinson Abbreviated Mental test.	Participants with the lowest dietary/plasma VitC status had the poorest cognitive function (OR: 1.6).	[3]
Clinical trials	Randomized open-label clinical trial: 23 AD patients receiving cholinergic treatment.	400 IU VitE and 1000 mg VitC per day or no vitamin treatment. CSF samples at baseline, one month and twelve months.	Clinical and neuropsychological assessment.	Significant increases in VitC content in CSF and decreases in autoxidation (*p* < 0.05). No neuropsychological differences.	[140]

Abbreviations: VitC, vitamin C; VitE, vitamin E; DHA, dehydroascorbic acid; Asc, ascorbate; VitA, vitamin A; AchE, Acethylcholine esterase; TAC, total antioxidant capacity; SOD, superoxide dismutase; MDA, malondialdehyde; SVCT, sodium-dependent vitamin C transporter; Aβ, beta-amyloid; AD, Alzheimer’s disease; CSF, cerebrospinal fluid; BMI, body mass index; ELISA, enzyme-linked immunosorbent assay; IP, intra-peritoneal; IHC, immunohistochemistry; FRAP, ferric reducing antioxidant capacity; MMSE, Mini Mental State Examination; CAMDEX, Cambridge Mental Disorders of the Elderly Examination; ADAS-cog, Alzheimer’s Disease Assessment Scale; TICS, Telephone Interview of Cognitive Status; CASI, Cognitive Abilities Screening Instrument; GMS, Geriatric Mental State; CDR, Clinical Dementia Rating; RR, relative risk; OR, odds ratio; SD, standard deviation.

### 4.3. Vitamin C and Stroke

Another disease that frequently leads to negative consequences on cognitive ability is stroke. Animal model studies have shown that brain VitC concentrations increase during ischemia, leading to speculations that VitC may play a neuroprotective role in these events [141]. In rodents and primates, VitC supplementation has been shown to limit the infarct area produced by middle cerebral artery occlusion (MCAO) both with and without subsequent reperfusion [51,52,142,143]. C57BL/6J mice subjected to transient or permanent MCAO and treated with administration of either ascorbate (250/500 mg/kg intravenous (IV)) or DHA (40/250/500 mg/kg IV) immediately before, 15 min after or 3 h after MCAO suggests a beneficial and dose-dependent effect of DHA on cerebral blood flow with subsequent reduced infarction-size and mortality, whereas ascorbate did not result in comparable effects [51].

In spontaneously hypertensive and stroke prone rats (SHR-SP), the expression of proteins such as glutathione S-transferase glutathione peroxidase are reportedly decreased together with brain total antioxidant capacity, while MDA is increased compared to spontaneously hypertensive rat (SHR) that are not stroke prone, indicating that increased oxidative stress may be a risk factor in the progression of stroke-related disease [52]. To further examine this, 24 h-MCAO was performed following four weeks of ascorbate 200 mg/kg) and VitE (100 mg/kg) supplementation PO once daily in both SHR-SP and SHR rats. The mean infarct area of SHR-SP was significantly larger than SHR with areas of 31.6% ± 5.4% and 23.0% ± 3.3%, respectively (*p* = 0.004) [52]. Treatment of SHR-SP rats with VitC and VitE decreased MDA, increased total antioxidant capacity and glutathione peroxidase activity and decreased the infarct area significantly, suggesting protective means of antioxidant treatment on oxidative stress and ischemia. Moreover, treatment of Macacca radiata monkeys with ascorbate (500 mg/kg; maximum of 2 grams, IV) before MCAO significantly reduced infarct size compared to control animals (7.3% ± 2.7%* vs.* 22.1% ± 6.7%; *p* = 0.0003), supporting a protective role of VitC in the amelioration of stroke induced damage [142].

Unfortunately, results from human studies have not been as consistent as those from animal studies. In a study of ischemic stroke patients receiving standard stroke treatment with or without VitC supplementation (500 mg/day IV) for ten days, no significant effect of VitC on National Institute of Health-Stroke Scale neurological status ten days or three months after the stroke incident could be detected [144]. However, compared to experimental studies in animals, human treatments are commenced at a much later stage following stroke-diagnosis, and may thus fail in targeting a potential narrow therapeutic window of VitC intervention. Another reason may be that the effect of VitC is more pronounced in the prevention of stroke in humans. Several large-scale human epidemiological studies have found an inverse relationship between plasma VitC and incidence of stroke suggesting that deficiency could be an important contributor to the development of disease [50,145,146,147]. An overview of studies on VitC and stroke in both experimental animal models and in humans is presented in Table 3.

**Table 3:** Vitamin C and stroke.

Species		Intervention	Measurement	Outcome	Reference
*In vivo studies*					
C57BL/6J mice.		DHA (40/250/500 mg/kg) or Asc (250/500 mg/kg) IV on three time points following MCAO.	Cortical cerebral blood flow, infarct volume, neurological assessment, mortality.	DHA improved cerebral blood flow dose-dependent. Decreased infarct size and mortality (*p* < 0.05). Asc did not show these effects.	[51]
SHR and SHR-SP rats (4–5 months old).		VitC (200 mg/kg) and VitE (100 mg/kg) PO once daily for 4 weeks MCAO of 24 h duration.	2D Western blot of antioxidative protein expression, TAC, GSH-Px and MDA in brain. Cerebral infarct area.	VitC + E treatment significantly reduced oxidative stress and infarct area in SHR-SP (*p* < 0.01).	[52]
Male Sprague-Dawley rats (4 weeks old) with or without STZ-induced diabetes for six weeks		VitC (100 mg/kg) PO once daily for 2 weeks following MCAO/Re	Infarct volume and edema, neurological score.	VitC treatment significantly reduced infarct area, edema and neurological score in both non-diabetic and diabetic animals compared to untreated controls (*p* < 0.01).	[143]
Maccaca radiata monkey.		Ascorbate (500 mg/kg up to 2 g IV) immediately before MCAO of 4 h duration.	Cerebral infarct area.	VitC treatment significantly reduced infarct area (*p* = 0.0003).	[142]
**Design and Subjects**		**Intervention**	**Measurements**	**Outcome**	**Reference**
*Clinical Studies*					
Cohort studies	Department of Health and Social Security nutritional survey: 730 participants age ≥65 years.	Food diary and interviews. Participants were followed for 20 years.	Plasma VitC, physical examination.	Participants in the highest third of VitC intake had a RR = 0.5, when compared with the lowest third.	[146]
	The Nurses’ Health Study: 85,118 participants age 30–55 years.	Semi-quantitative questionnaire on lifestyle, supplemental use and medical history. The participants were followed for 16 years.		VitC supplemental use is significantly associated with lower risk of coronary heart disease (RR = 0.72).	[147]
	Cancer-Norfolk prospective study: 20,649 participants age 40–79 years.	Health and lifestyle questionnaire, socioeconomic data	Physical examination, plasma VitC content.	Plasma VitC was inversely related to risk of stroke. Participants in top quantile had a RR = 0.58.	[145]
	Basel prospective study 2974 men	The participants were followed for 12 years.	Baseline values of VitC and β-carotene in plasma.	Low levels of VitC and β-carotene were related to an increased risk of dying from ischemic heart disease or stroke.	[50]
Clinical trials	Double-blind randomized clinical trial: 40 patients (0–2 years after cardiac transplant).	500 mg VitC and 400 IU VitE twice daily for one year or placebo.	Plasma VitC and VitE content. Average intimal index, coronary endothelium-dependent vasoreactivity.	Supplementation with VitC + E caused retardation of early signs of atherosclerosis associated with heart transplantation (*p* = 0.008).	[148]
	Sixty ischemic stroke patients (72.8 ± 10.4 years), VitC *vs.* non-VitC group and 20 controls (69.8 ± 10.5 years).	500 mg/day VitC IV in addition to standard stroke treatment for ten days starting the day after stroke.	NIHSS neurological status and bilirubin, creatinine, uric acid, and TAC day one, three, five and ten of treatment. NIHSS three months after stroke.	No difference in clinical status of patients during the ten day treatment or after the three months follow-up.	[144]

Abbreviations: VitC, vitamin C; VitE, vitamin E; DHA, dehydroascorbic acid; Asc, Ascorbate; TAC, total antioxidant capacity; MDA, malondialdehyde; GSH-Px, glutathione peroxidase; MCAO, middle cerebral artery occlusion; IV, intra-venous; PO, per os; NIHSS, National Institutes of Health Stroke Scale; SHR, spontanous hypertensive rat; SHR-SP, spontaneously hypertensive rat stroke prone; RR, relative risk; STZ, streptozotocin.

## 5. Conclusions

Collectively, controlled experimental animal studies support VitC as a key factor in the prevention of cognitive decline following both aging associated alteration as well as neurodegenerative disorders. Data also supports a direct effect of VitC deficiency on brain function particularly during development and/or regeneration following traumatic brain injury such as ischemic insults. Reports from large population surveys in humans point to VitC deficiency as contributing factor in disease propagation, however, randomized controlled clinical trials have not been able to confirm the putative beneficial effects of VitC supplementation and/or intervention. A possible contributing reason for this apparent discrepancy may include differences in inclusion criteria, typically in recording of VitC status prior to study start, admitting individuals already saturated in VitC and thus unlikely to experience any effects of further supplementation. Moreover, the common use of multivitamins or combinations of vitamins in the intervention studies often precludes the ability to isolate the effects of the individual substances. Consequently, further randomized controlled trials using VitC as a single substance are required to elaborate on whether findings from experimental models translate into effects in humans, possibly focusing on specific subgroups with increased prevalence of VitC deficiency, as well as the identification of potential therapeutic windows.
